# Gut microbiome and mycobiome in inflammatory bowel disease patients with *Clostridioides difficile* infection

**DOI:** 10.3389/fcimb.2023.1129043

**Published:** 2023-02-06

**Authors:** Si Yu, Xiaomeng Ge, Hui Xu, Bei Tan, Bowen Tian, Yujie Shi, Yimin Dai, Yue Li, Songnian Hu, Jiaming Qian

**Affiliations:** ^1^ Department of Gastroenterology, Peking Union Medical College Hospital, Chinese Academy of Medical Sciences & Peking Union Medical College, Beijing, China; ^2^ Microbial Resource and Big Data Center, Institute of Microbiology, Chinese Academy of Sciences, Beijing, China; ^3^ State Key Laboratory of Microbial Resources, Institute of Microbiology, Chinese Academy of Sciences, Beijing, China; ^4^ University of Chinese Academy of Sciences, Beijing, China

**Keywords:** inflammatory bowel disease, *Clostridioides difficile* infection, metagenomics, gut microbiome, mycobiome

## Abstract

**Background:**

*Clostridium difficile* infection (CDI) is common in patients with inflammatory bowel disease (IBD) and has been reported as a risk factor for poor outcome. However, gut microbiome and mycobiome of IBD patients with CDI have been barely investigated. This study aimed to assess the gut microbiome and mycobiome in IBD patients with CDI.

**Methods:**

We collected fecal samples from patients with active IBD and concomitant CDI (IBD-CDI group, n=25), patients with active IBD and no CDI (IBD-only group, n=51), and healthy subjects (HC, n=40). Patients’ characteristics including demographic data, disease severity, and medication history were collected. Metagenomic sequencing, taxonomic and functional analysis were carried out in the samples.

**Results:**

We found that the bacterial alpha diversity of the IBD-CDI group was decreased. The bacterial and fungal beta diversity variations between IBD patients and HC were significant, regardless of CDI status. But the IBD-CDI group did not significantly cluster separately from the IBD-only group. Several bacterial taxa, including *Enterococcus faecium*, *Ruminococcus gnavus*, and *Clostridium innocuum* were overrepresented in the IBD-CDI group. Furthermore, IBD patients with CDI were distinguished by several fungal taxa, including overrepresentation of *Saccharomyces cerevisiae*. We also identified functional differences in IBD patients with CDI include enrichment of peptidoglycan biosynthesis. The network analysis indicated specific interactions between microbial markers in IBD-CDI patients.

**Conclusion:**

IBD patients with CDI had pronounced microbial dysbiosis. Gut micro-ecological changes in IBD patients with CDI might provide insight into the pathological process and potential strategies for diagnosis and treatment in this subset of patients.

## 1 Introduction

Inflammatory bowel disease (IBD) is a chronic inflammatory disorder of the gastrointestinal tract, including two main subtypes, ulcerative colitis (UC) and Crohn’s disease (CD). Dysbiosis of gut microflora is well recognized as an important factor in the pathogenesis and development of IBD (Pickard et al., 2017; Franzosa et al., 2019). *Clostridioides difficile* (*C. difficile*) is a Gram-positive, sporulating, anaerobic bacillus, widely distributed in human gut as an opportunistic pathogen (Czepiel et al., 2019). *C. difficile* infection (CDI) was reported to be the major cause of healthcare-associated infections around the world, and was also strongly associated with distinct alternations in the gut microbiota (Lamendella et al., 2018; Rodríguez et al., 2020; Berkell et al., 2021). Severe and recurrent CDI patients can benefit from gut microbiota reconstruction by fecal microbiota transplantation (FMT) (Hvas et al., 2019). IBD with concomitant CDI is a common condition with a prevalence up to 32% (Rodríguez et al., 2020). IBD patients with CDI were demonstrated to have more severe disease course, poor outcome, and an increase in morbidity and mortality (D’Aoust et al., 2017; Li et al., 2018).

Recent studies have tried to investigate the gut microbiome and in IBD patients with CDI. Dysbiotic microbiome and alternations of certain gut microorganisms were revealed (Sokol et al., 2018; Bushman et al., 2020; Hellmann et al., 2020; Mahnic et al., 2022). However, there is still a limited number of studies exploring the composition and changes of gut microbiome in IBD patients with CDI. Besides, most of these studies were performed with 16S rRNA gene sequencing, while metagenomic data could provide both the taxonomic and functional information in high resolution (Maccaferri et al., 2011). In addition, all the current studies have been focused on bacterial dysbiosis, while there is more and more evidence that nonbacterial microbes, such as fungi, might play unique and important roles in IBD pathogenesis and disease activity. Knowledge of the gut mycobiome might provide new insights for IBD therapeutic strategies and prognosis prediction (Chehoud et al., 2015; Sokol et al., 2017; Imai et al., 2019; Lemoinne et al., 2020; Frau et al., 2021; Ventin-Holmberg et al., 2021; Guzzo et al., 2022; Underhill and Braun, 2022; Li et al., 2022b). Nevertheless, gut mycobiome in IBD patients with CDI have never been described before.

Therefore, we conducted the study with the following aims: (1) to investigate the taxonomic and functional alternations of gut microbiome in IBD patients with CDI with metagenome sequencing; (2) to describe the gut mycobiome in IBD patients with CDI.

## 2 Materials and methods

### 2.1 Study design and sample collection

All participants were consecutively recruited from IBD patients who were hospitalized for active disease in Peking Union Medical College Hospital (Beijing, China) from November 2017 to May 2020. Diagnosis of IBD was established based on the clinical, radiological, endoscopic and histological criteria. Diagnosis of CDI was based on the presence of diarrhea and positive results of toxin A/B enzyme immunoassays and/or nucleic acid amplification test (Kucharzik et al., 2021). Participants who had taken metronidazole and/or vancomycin within a month before index hospitalization were excluded. Patients who had received fecal microbiota transplantation and/or colectomy were also excluded. Healthy volunteers who were physically fit and did not have any gastrointestinal disease, infectious disease, or other chronic disease were recruited as the healthy controls. Fecal samples of patients were collected once a patient was hospitalized and had received neither any antibiotics for CDI nor escalated medication for IBD. Fecal samples were collected in sterile collection tubes and were immediately transferred to a −80°C freezer for further analysis. Research approval was obtained from the Ethics Committees of Peking Union Medical College Hospital (approval no. JS-1494). All patients provided informed consent. The study conformed with the principles in the Declaration of Helsinki.

### 2.2 Shotgun sequencing and metagenomic analyses

Metagenomic DNA libraries were sequenced on Illumina platform and 150 bp paired-end reads were generated. Low-quality reads were removed with Trimmomatic (Bolger et al., 2014). Low-quality samples with a high proportion of low-quality reads and human-contaminating reads were removed in further analysis. Taxonomic classifications were assigned to metagenomic reads using MetaPhlAn3 (Beghini et al., 2021). Functional profile was obtained using HUMAnN2 (Abubucker et al., 2012). Discriminative microbial markers in each study group was identified with linear discriminant analysis effect size (Lefse) (Segata et al., 2011). Comparison and identification of differential pathways were performed using STAMP (Parks et al., 2014). Ecological network of gut microbiota in the three groups was analyzed using SparCC (Friedman and Alm, 2012). Cytoscape (version 3.9.1) was used for visualization of the networks.

### 2.3 Statistical analysis

R (version 4.2.1) was used for statistical analysis. Continuous variables with a non-Gaussian distribution are presented as the median and interquartile range (IQR) and were compared using the Mann-Whitney U test. Categorical data are presented as counts and percentages and were compared using chi-square test or Fisher’s exact test. Multiple group statistics was performed using Kruskal- Wallis test, and the *post-hoc* test was performed with Dunn’s multiple comparison test. The p values were corrected using the Benjamini and Hochberg procedure to control for the false discovery rate. *P* < 0.05 was considered to be significant.

## 3 Results

### 3.1 Clinical characteristics

A total of 169 participants including 129 IBD patients and 40 healthy controls were recruited. and 116 of them were included in the study population after quality control. The study population consist of three groups: patients with IBD and no CDI (IBD-only, n = 51), patients with IBD and CDI (IBD-CDI, n = 25), and healthy subjects (HC, n = 40). Clinical characteristics of the patients were shown in **Supplementary Table 1**. 50 patients were diagnosed with UC (65.8%), and 26 patients were diagnosed with CD (34.2%). Distribution of the diagnostic types was comparable between the IBD-CDI and IBD-only groups (**Supplementary Table 1**, p = 0.588). A majority of patients had moderate-to-severe disease (76.6% of the UC patients according to the Truelove-Witts criteria; 72.0% of the CD patients according to the Crohn disease activity index). Most patients had prior 5-aminosalicylic acid exposure (77.0%), while only a fraction of patients had been exposed to corticosteroid (36.8%), immunosuppressant (17.1%), biologics (14.5%), and antibiotics (18.4%) within a month before the index hospitalization. The two groups of patients were comparable, notably in terms of age, lifestyles, body mass index, diagnostic subtype, disease severity, laboratory data, and medication history. However, patients in the IBD-only group were more frequently male, and had more often perianal lesion than patients in the IBD-CDI group.

### 3.2 Gut microbiome profiles of the study groups

**Figure 1:**
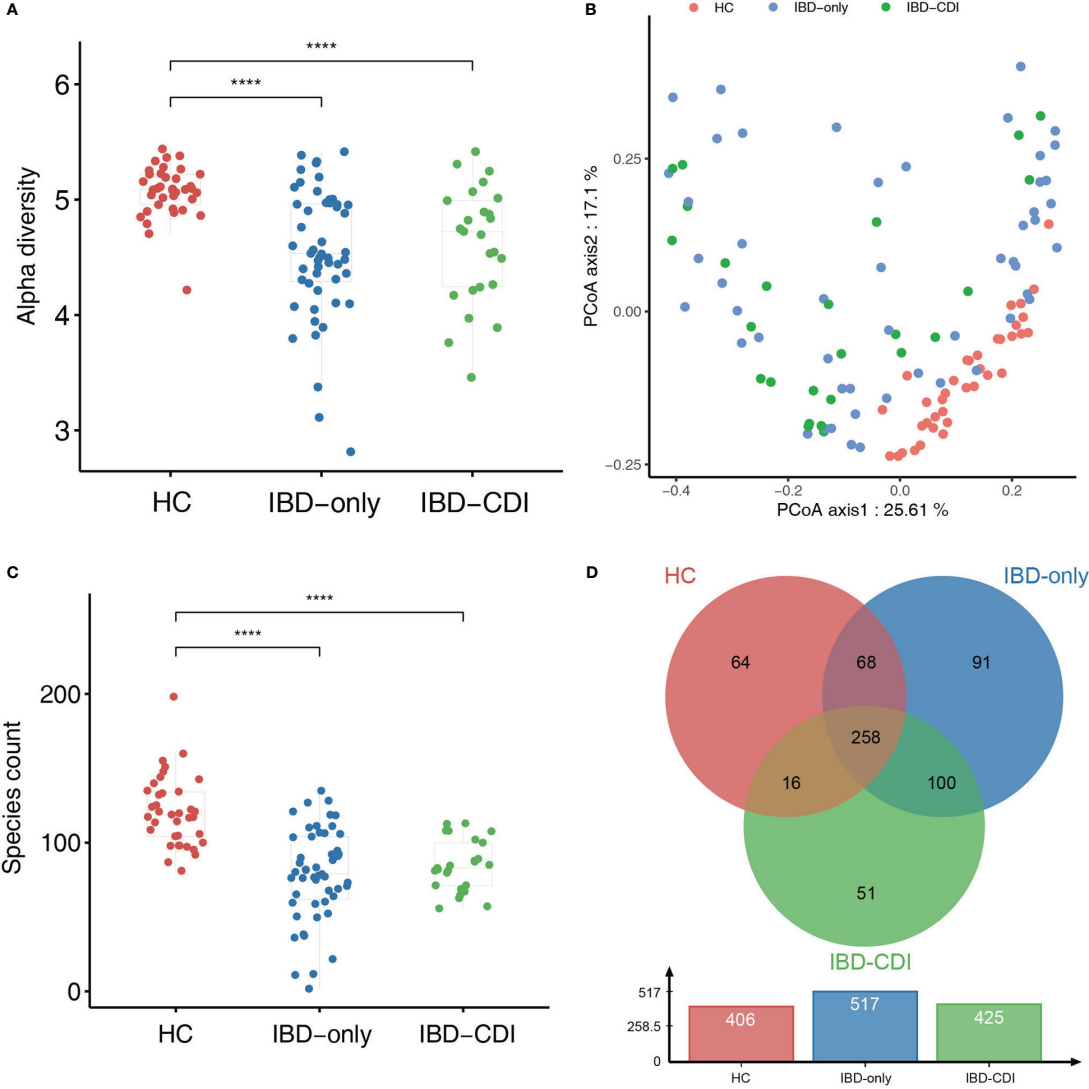
Biodiversity of gut microbiome in the HC, IBD-only, and IBD-CDI groups. **(A)** The alpha diversity was measured in the Shannon index. **(B)** PCoA plots of the three study groups. **(C)** Box plots of the number of species among the three groups. **(D)** The venn diagram of the common and unique species among the three groups. ****P < 0.0001.

Compared with HC samples, the alpha diversity (assessed by the Shannon index) was significantly decreased in IBD-only and IBD-CDI groups. No significant difference of alpha diversity was observed between IBD-only and IBD-CDI groups (**Figure 1A**). The beta diversity was significantly different among all IBD patients compared with controls (assessed by the Bray-Curtis dissimilarity index, PERMNOVA *P* = 0.001). However, the beta diversity did not show a clustering of samples according to *C. difficile* infection status (**Figure 1B**). At the species level, the species counts of gut microbiota were decreased in both the IBD-only and IBD-CDI groups, compared with HC (**Figure 1C**). There were 258 common species among the three groups (**Figure 1D**; **Supplementary Table 2**).

**Figure 2:**
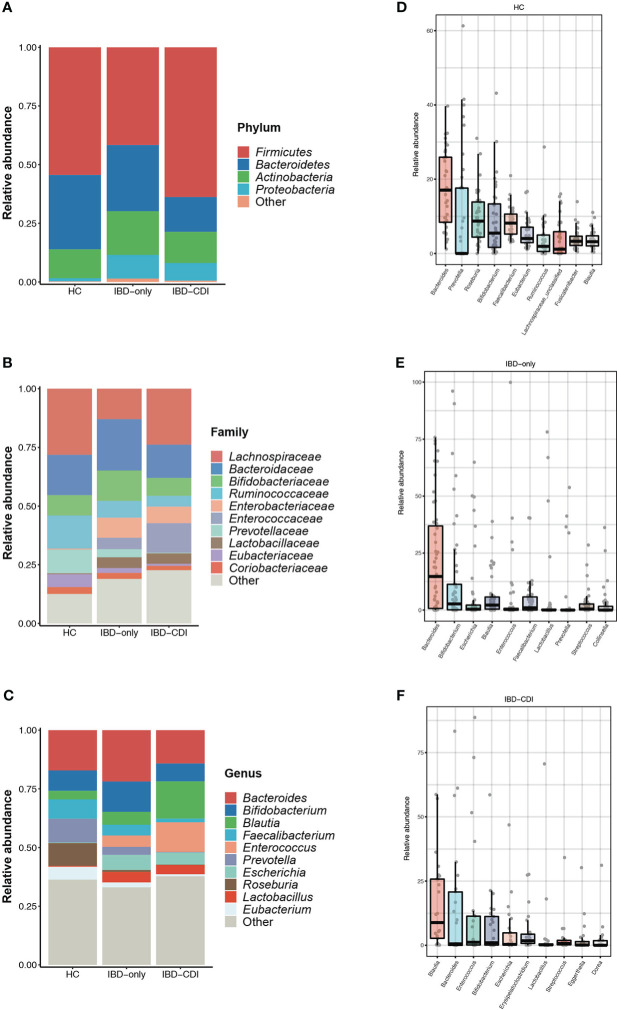
Gut microbiome composition in the HC, IBD-only, and IBD-CDI groups. **(A-C)** Global composition at the phylum, family, and genus level. **(D-F)** Box plots of the top 10 genera sorted by the mean values of relative abundance of the three groups.

In the three study groups, the gut microbiome was dominated by *Firmicutes*, *Bacteroidetes*, *Actinobacteria*, and *Proteobacteria* at the phylum level (**Figure 2A**; **Supplementary Table 3**), and *Lachnospiraceae*, *Bacteroidaceae*, *Bifidobacteriaceae*, *Ruminococcaceae*, *Enterobacteriaceae*, *Enterococcaceae*, and *Prevotellaceae* at the family level (**Figure 2B**; **Supplementary Table 3**). The most dominant genera identified were *Bacteroides*, *Bifidobacterium*, *Blautia*, *Faecalibacterium*, *Enterococcus*, *Prevotella*, *Escherichia*, *Roseburia*, *Lactobacillus*, and *Eubacterium* (**Figure 2C**; **Supplementary Table 3**). Specifically, relative abundance of *Faecalibacterium* and *Bacteroides* were decreased, and the *Enterococcus* and *Blautia* abundance were increased in the IBD-CDI group compared with the other two groups. The HC group had higher relative abundance of *Roseburia* compared with the other two groups of patients (all *P* < 0.05, **Figures 2D–F**; **Supplementary Table 3**).

**Figure 3:**
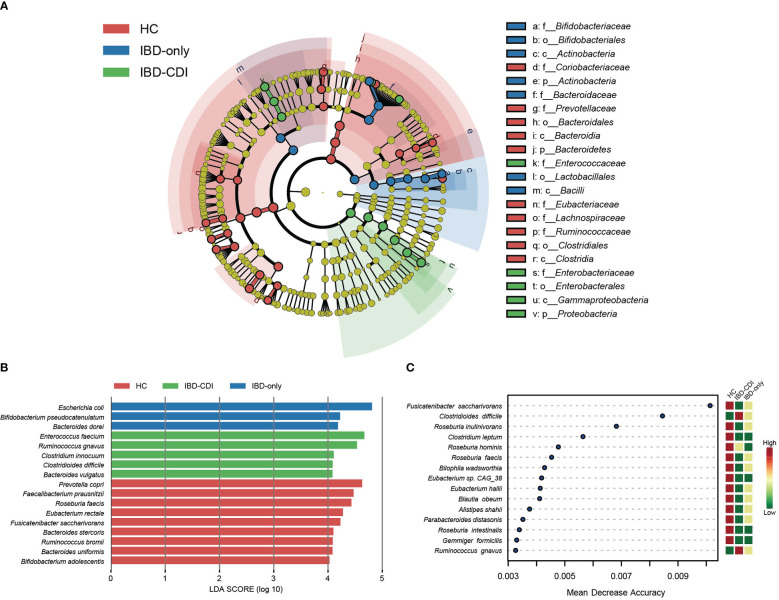
Microbial marker analysis. **(A)** The cladogram plot of the three study groups. **(B)** LDA score distribution bar plot for marker species. Species with LDA score >4.0 and *P* < 0.05 were regarded as significantly discriminative markers. **(C)** Analysis with the machine learning algorithm random forest. Mean decrease accuracy indicated the importance of the markers. Only taxa present in >20% of the samples were taken into account.

Microbial markers among healthy control and IBD patients with or without CDI were identified with lefse analysis (**Figure 3**). According to the cladogram plot, the differential taxa in the IBD-CDI group included the phylum *Proteobacteria*, the order *Enterobacterales*, the class *Gammaproteobacteria*, and the family *Enterococcaceae* and *Enterobacteriaceae* (**Figure 3A**; **Supplementary Table 4**). At the species level, the IBD-CDI group was characterized by the enrichment of *Enterococcus faecium (E. faecium)*, *Ruminococcus gnavus (R. gnavus)*, *Clostridium innocuum (C.innocuum)*, *C. difficile*, and *Bacteroides vulgatus* (**Figure 3B**; **Supplementary Table 4**). The random forest classifier was used to identify the species that best discriminated the three groups of samples from each other. As shown in **Figure 3C**, the two species enriched in the IBD-CDI group, *C. difficile* and *R. gnavus*, were identified as two important microbial markers.

### 3.3 Gut mycobiome alternations in IBD patients with CDI

**Figure 4:**
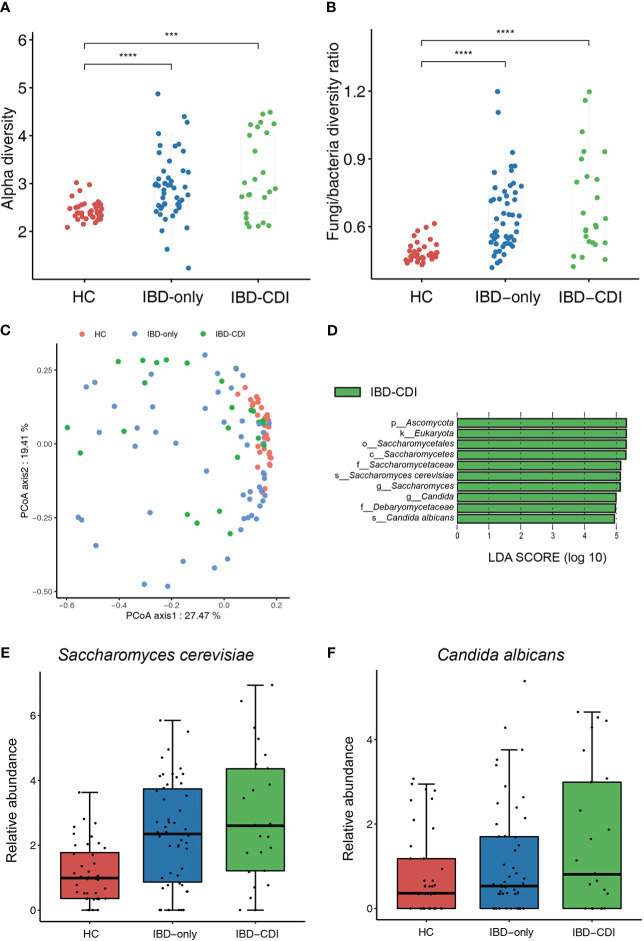
Gut mycobiome alternations among the HC, IBD-only, and IBD-CDI groups. **(A)** The fungal alpha diversity. **(B)** Fungi-to-bacteria alpha diversity ratio. **(C)** Fungal beta diversity analysis with the PCoA plots of the three study groups. **(D)** Lefse analysis of the mycobiome alterations. Taxa with LDA score >4.0 and *P* < 0.05 were regarded to be significantly discriminative. Only taxa present in >20% of the samples were taken into account. **(E)** Box plot of relative abundance of *Saccharomyces cerevisiae*. **(F)** Box plot of relative abundance of *Candida albicans*. ***P < 0.001. ****P < 0.0001.

We then assessed the gut fungal microbiota to identify the mycobiome alternations in IBD patients with CDI. The alpha diversity of fungi in the IBD-only and IBD-CDI groups was significantly higher than that in the HC group. The analysis of alpha diversity showed no difference between IBD-only and IBD-CDI groups (**Figure 4A**; **Supplementary Table 5**). To further explore the equilibrium between bacteria and fungi diversity in the gut, we calculated the fungi-to-bacteria diversity ratio. This ratio was significantly increased in patients with IBD-only or IBD-CDI compared with HC. Similarly, no distinct difference was observed between the IBD-only and IBD-CDI groups (**Figure 4B**). A significant difference of fungal beta diversity was observed between samples from HC and patients with IBD-only or IBD-CDI (**Figure 4C**). The results of lefse analysis showed several mycobiome alterations associated with IBD-CDI, including the enrichments of the order *Saccharomycetales*, the class *Saccharomycetes*, the families *Saccharomycetaceae* and *Debaryomycetaceae*, the genera *Saccharomyces* and *Candida*, and the species *Saccharomyces cerevisiae* (*S. cerevisiae*) and *Candida albicans (C. albicans)* (**Figure 4D**; **Supplementary Table 5**). As showed in **Figures 4E, F**, the relative abundance of *Saccharomyces cerevisiae* and *Candida albicans* increased in the sequence of HC, IBD-only, and IBD-CDI.

### 3.4 Functional signatures of IBD patients with CDI

**Figure 5:**
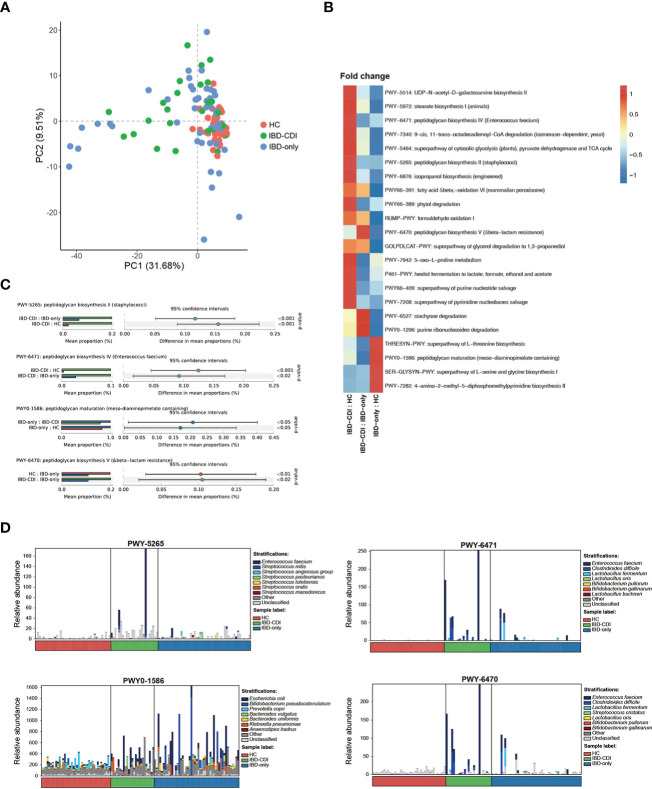
Functional analysis**(A)** Principal component analysis of the functional composition. **(B)** Fold changes of the significantly different pathways between IBD-CDI and IBD-only groups. Only pathways present in >20% of the samples were taken into account. Data was normalized with z score in the heatmap. **(C)** Bar chart of the four pathways involved with peptidoglycan metabolism. **(D)** Stratified analysis of the four pathways by species.

Functional analysis of the three study groups was conducted with HUMANn2. Principal component analysis (PCA) was used to demonstrate the functional composition. The HC samples clustered together, while the IBD-only and IBD-CDI samples were widely scattered (**Figure 5A**). A total of 275 differential pathways among the HC, IBD-only, and IBD-CDI groups were identified (**Supplementary Table 5**). In *post-hoc* analysis, 22 pathways with significantly different relative abundance between IBD-only and IBD-CDI were identified (**Figure 5B**; **Supplementary Table 5**). Four of these pathways were related to peptidoglycan metabolism: PWY-5265 (peptidoglycan biosynthesis II [staphylococci]), PWY-6471 (peptidoglycan biosynthesis IV [Enterococcus faecium]), PWY0-1586 (peptidoglycan maturation [meso-diaminopimelate containing]), and PWY-6470 (peptidoglycan biosynthesis V [beta-lactam resistance]). The mean relative abundance of PWY-5265 and PWY-6471 in patients with IBD-CDI were higher than those with IBD-only or HC. Patients with IBD-CDI had decreased abundance of PWY0-1586 and increased abundance of PWY-6470 compared with patients with IBD-only (**Figure 5C**). We further conducted stratified analysis of these four pathways to identify the metagenomically contributed species (**Figure 5D**; **Supplementary Table 5**). *Enterococcus faecium* was the dominant contributor in PWY-5265 in the IBD-CDI group. Additionally, *Enterococcus faecium* and *Clostridioides difficile* were the main contributors in PWY-6470 and PWY-6471 in these patients. PWY0-1586 were not dominated by any single species.

### 3.5 Correlation networks of the study groups

**Figure 6:**
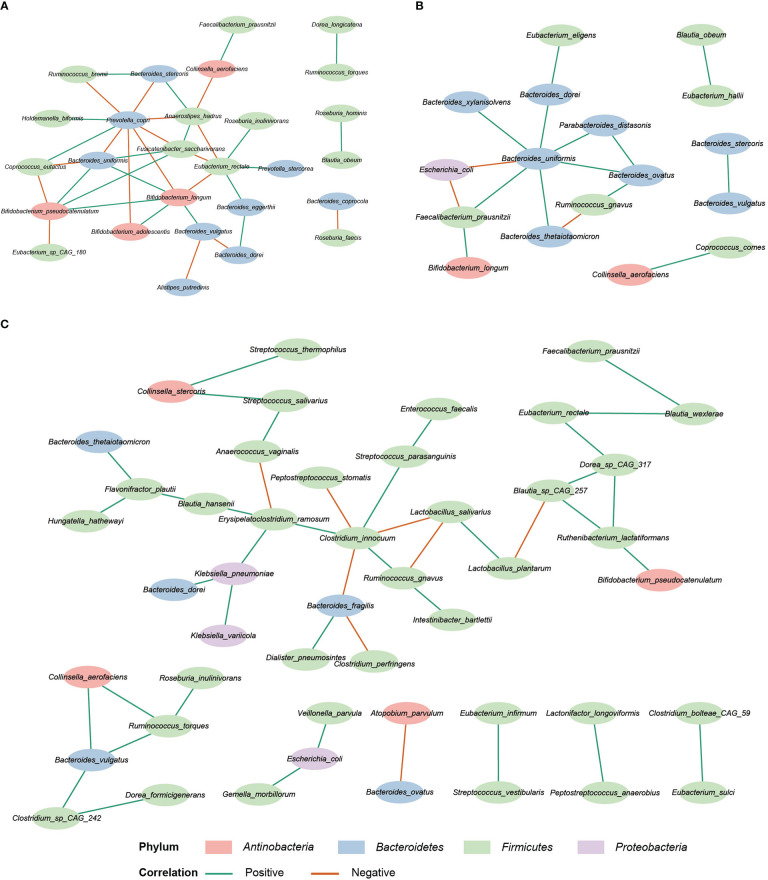
Correlation network analysis. **(A)** The HC network. **(B)** The IBD-only network. **(C)** The IBD-CDI network. Only species present in >20% of the samples were taken into account, and only the statistically significantly correlations were shown.

SparCC analysis was used to construct the correlation network of the three study groups (Friedman and Alm, 2012). Compared with HC and IBD-only, the network of IBD-CDI had more nodes and edges. But the HC network had higher average number of neighbors than the IBD-only and IBD-CDI networks (**Figure 6**; **Supplementary Table 6**). Several microbial markers were present in the network, such as *Ruminococcus gnavus* and *Clostridium innocuum*. *Ruminococcus gnavus* was present in both the IBD-only and IBD-CDI network. In the IBD-only network, it was positively correlated with *Bacteroides ovatus*, and was negatively correlated with *Bacteroides thetaiotaomicron*. In the IBD-CDI network, it was positively related with *Clostridium innocuum* and *Intestinibacter bartlettii*, and was negatively correlated with *Lactobacillus salivarius (L. salivarius)*. *Clostridium innocuum* was presented in the center of the IBD-CDI network, and was also negatively correlated with *Lactobacillus salivarius*.

## 4 Discussion

CDI is a common complication of IBD. *C. difficile* coinfection is associated with longer hospital stay, escalated medication, and higher colectomy rates of IBD patients. Investigation of gut microecology in IBD patients with CDI may facilitate the understanding of the disease, and provide new perspectives for treatments. In this study, we showed that IBD-CDI patients had a pronounced microbial dysbiosis and specific alternations in the gut mycobiome and functional profile. Bacterial dysbiosis in gut of IBD patients with CDI, including decreased alpha diversity and altered composition, were consistent with previous reports (Sokol et al., 2018; Mahnic et al., 2022). In keeping with previous results, clustering of the samples in IBD-only and IBD-CDI indicated heterogeneity regardless of *C. difficile* coinfection status (Bushman et al., 2020). In addition to confirmation, new data about the bacterial microbiota alternations were also provided. We found a decreased proportion of *Faecalibacterium* and an increased proportion of *Enterococcus* in IBD patients with CDI compared with IBD-only. *Faecalibacterium* has been reported as a probiotic, which had anti-inflammatory potential *via* butyrate production and regulation of butyrate-mediated inflammatory pathways (Roychowdhury et al., 2018; Zhou et al., 2018; Xu et al., 2020; Li et al., 2022a). Depletion of *Faecalibacterium* has been observed in pediatric CD patients with CDI (Hellmann et al., 2020). In our study, relative abundance of *Faecalibacterium* was significantly decreased in IBD-only patients compared with HC, and this alternation was even enhanced when comparing IBD-CDI and HC, suggesting a more drastic micro-ecological changes with both medical complications.

Several studies have shown that gut mycobiome shifts existed between IBD patients and healthy population. For the first time, we report the mycobiome alternations in IBD patients with CDI. Gut mycobiome in IBD patients with CDI was characterized by an increased alpha diversity compared with healthy controls, and by certain taxonomic alternations, including a markedly increased abundance of *S. cerevisiae*. Chehoud et al. have reported decreased fungal alpha diversity in IBD patients (Chehoud et al., 2015). However, more recent studies reported no significant difference between IBD patients and healthy people in fungal diversity (Imai et al., 2019; Lemoinne et al., 2020; Li et al., 2022b). Furthermore, primary sclerosing cholangitis, another common comorbidity of IBD, was reported with elevated biodiversity compared with healthy people (Lemoinne et al., 2020). In accordance with previous studies, we found that the fungi-to-bacteria diversity ratio was decreased in IBD patients compared with HC (Sokol et al., 2017; Lemoinne et al., 2020), suggesting disturbed balance between fungal and bacterial flora. *S. cerevisiae* has long been correlated with IBD, since the anti-S. cerevisiae antibodies (ASCA) were specifically elevated in CD patients and might help in diagnosis and outcome prediction of CD patients (Main et al., 1988; Quinton et al., 1998; Forcione et al., 2004; Reese et al., 2006). *S. cerevisiae* was detected in the fecal samples of pediatric CD patients (Lewis et al., 2015), and its abundance was decreased in UC patients and active IBD patients compared with healthy controls (Sokol et al., 2017; Li et al., 2022b). Overall, current knowledge of gut mycobiome is limited, and the results remain inconsistent. Whether gut mycobiome contributes to pathogenesis and development of IBD-CDI remains to be explore.

In the present study, we demonstrated that *E. faecium* was one of the differential microbial markers in IBD patients with CDI, and was involved in the peptidoglycan metabolism pathways, which were enriched in this group of patients. Both *E. faecium* and *C. difficile* were nosocomial pathogens and shared similar risk factors (Gerding, 1997). As reported, vancomycin-resistant *E. faecium* was detected in around 17%~19% patients with CDI (Garbutt et al., 1999; Ozsoy and Ilki, 2017). Vancomycin-resistant *E. faecium* colonization was reported as a risk factor of *C. difficile*-associated disease in immunocompromised population (Trifilio et al., 2013). Glycopeptide-resistant *E. faecium* can produce modified peptidoglycan precursors, which block the target of the antimicrobial agents (Hendrickx et al., 2013). However, there is also studies indicating that some commensal strains of *E. faecium* can facilitate probiotic bacteria activity against *C. difficile* pathogenesis by secreting peptidoglycan hydrolase and enhancing host immunity (Zheng et al., 2016; Kim et al., 2019). Moreover, this peptidoglycan remodeling activity might also benefit patients receiving immunotherapy (Griffin et al., 2021). In addition to peptidoglycan metabolism, previous study has suggested that both *E. faecium* and *C. difficile* could affect the antibiotic resistant activities of the antimicrobial resistant (Kunishima et al., 2019).

The other two microbial markers in IBD patients with CDI, *C.innocuum* and *R. gnavus*, were also implied in the network analysis. *C.innocuum* is a gram-positive, spore-forming, anaerobic bacterium (Cherny et al., 2021). *C.innocuum* frequently coexist with *C. difficile*, a previous study indicated that *C.innocuum* might cause antibiotic-associated diarrhoea with clinical manifestation similar to CDI (Chia et al., 2018; Cherny et al., 2022). Besides, *C.innocuum* can lead to creeping fat in Crohn’s disease (Ha et al., 2020), and is associated with poor clinical outcomes in UC patients (Le et al., 2022). Another biomarker, *R. gnavus*, was positively correlated with *C.innocuum* in IBD-CDI network. *R. gnavus* can utilize the mucin and fucosylated glycans in the gastrointestinal tract, and it can also produce an inflammatory polysaccharide, which might induce secretion of tumor necrosis factor alpha by dendritic cells (Crost et al., 2013; Henke et al., 2019; Henke et al., 2021). In addition, high levels of *R. gnavus* were observed in the donors of UC patients who failed the FMT, confirming a proinflammatory role of *R. gnavus* in the gut of IBD patients (Fuentes et al., 2017). Notably, both *C.innocuum* and *R. gnavus* were negatively correlated with *L. salivarius*, a long recognized probiotics. Previous studies showed that probiotic strategies containing *L. salivarius* had beneficial effects on IBD patients (Palumbo et al., 2016; Ghavami et al., 2020). A recent study suggested that DL-endopeptidase-producing *L. salivarius* could promote the restoration of the pattern-recognition receptor NOD2 in mice with CD, and further exerted potent anti-colitis effects (Gao et al., 2022). Therefore, probiotic supplement with *L. salivarius* and regulation of the balance between *C.innocuum*, *R. gnavus*, and *L. salivarius* might be potential therapeutic options for IBD patients with CDI.

This study has several limitations. First, it is a single-centre study, with relatively small sample size, which may introduce bias about the composition and alternations of gut microflora. But our sample size is larger than former studies in IBD patients with CDI (Sokol et al., 2018; Bushman et al., 2020). Second, the study is cross-sectional, samples were only collected before the initiation of treatment. Therefore, this study cannot provide information about dynamics of gut microbiota. Longitudinal analysis is warranted in the future. Lastly, the study population were heterogenous, and the prior medication exposure varied among patients. Future analysis to address these limitations would be useful.

In summary, this study showed that IBD patients with CDI had pronounced microbial dysbiosis, with decreased biodiversity and certain bacterial and fungal alternations. Several functional changes, such as peptidoglycan metabolism, were enriched in this subset of patients. We also found signatures of interactions between microbial markers in these patients, which might provide new insights for microbiota-based treatment in IBD-CDI patients.

## Data availability statement

The original contributions presented in the study are included in the article/**Supplementary Material**, further inquiries can be directed to the corresponding author/s.

## Ethics statement

The studies involving human participants were reviewed and approved by The Ethics Committees of Peking Union Medical College Hospital. The patients/participants provided their written informed consent to participate in this study.

## Author contributions

SY, XG, and HX contributed equally to this study. YL and HX conceived and designed the study. SY, XG, HX, BWT, YS, BT, and YD performed the experiments. SY, XG, and HX analysed the data and wrote the manuscript. YL, SH, and JQ provided general guidance and revised the manuscript. All authors contributed to the article and approved the submitted version.

## Supplementary material

Supplementary Table 1 Clinical profile of the patients

Supplementary Table 2 Species list of the study groups

Supplementary Table 3 Taxonomy composition of the study groups

Supplementary Table 4 Microbial markers with significant differences among the study groups

Supplementary Table 5 Gut mycobiome analysis of the study groups

Supplementary Table 6 Functional analysis of the study groups

Supplementary Table 7 Network analysis of the study groups
