# Supplementary material for: Gut microbiome and mycobiome in inflammatory bowel disease patients with Clostridioides difficile infection
Source: Front Cell Infect Microbiol. 2023 Feb 6;13:1129043. doi: 10.3389/fcimb.2023.1129043 (PMC9940757; doi:10.3389/fcimb.2023.1129043)
Supplement: Supplementary Table 1 — Clinical profile of the patients [file Table_1.docx]

Supplementary Material

**Gut microbiome and** **mycobiome in** **inflammatory bowel disease patients with** ***Clostridium difficile* infection**

**Si Yu, Xiaomeng Ge****, Hui Xu, Bei Tan, Bowen Tian, Yujie Shi, Yimin Dai, Songnian Hu, Yue Li^*^, Jiaming Qian**

*** Correspondence:** Yue Li: liyue@pumch.ac.cn

# Supplementary Figures and Tables

## Supplementary Tables

**Supplementary Table 1.** Clinical profile of the patients.

|  | **ALL** | **IBD-only** | **IBD-CDI** | **P value** |
| --- | --- | --- | --- | --- |
|  | ***N=76*** | ***N=51*** | ***N=25*** |  |
| **Gender** |  |  |  | 0.030 |
| female | 28 (36.8%) | 14 (27.5%) | 14 (56.0%) |  |
| male | 48 (63.2%) | 37 (72.5%) | 11 (44.0%) |  |
| **Age, y** | 40.0 [28.0;52.0] | 38.0 [28.0;50.5] | 43.0 [30.0;53.0] | 0.319 |
| **Hospital stay, d** | 16.0 [8.00;22.0] | 14.0 [8.00;20.0] | 19.0 [11.8;23.5] | 0.151 |
| **Smoking** |  |  |  | 0.569 |
| cessation | 9 (12.0%) | 6 (11.8%) | 3 (12.5%) |  |
| no | 58 (77.3%) | 38 (74.5%) | 20 (83.3%) |  |
| yes | 8 (10.7%) | 7 (13.7%) | 1 (4.17%) |  |
| **Drinking** |  |  |  | 1.000 |
| cessation | 10 (13.3%) | 7 (13.7%) | 3 (12.5%) |  |
| no | 61 (81.3%) | 41 (80.4%) | 20 (83.3%) |  |
| yes | 4 (5.33%) | 3 (5.88%) | 1 (4.17%) |  |
| **Diagnosis** |  |  |  | 0.588 |
| CD | 26 (34.2%) | 19 (37.3%) | 7 (28.0%) |  |
| UC | 50 (65.8%) | 32 (62.7%) | 18 (72.0%) |  |
| **Montreal classification of UC** | | | | 1.000 |
| E2 | 10 (20.4%) | 7 (21.9%) | 3 (17.6%) |  |
| E3 | 39 (79.6%) | 25 (78.1%) | 14 (82.4%) |  |
| **Truelove Witts criteria of UC** | |  |  | 0.619 |
| mild | 11 (23.4%) | 6 (20.0%) | 5 (29.4%) |  |
| moderate | 29 (61.7%) | 20 (66.7%) | 9 (52.9%) |  |
| severe | 7 (14.9%) | 4 (13.3%) | 3 (17.6%) |  |
| **Mayo Score of UC** | |  |  | 0.793 |
| 1 | 10 (23.3%) | 6 (23.1%) | 4 (23.5%) |  |
| 2 | 18 (41.9%) | 12 (46.2%) | 6 (35.3%) |  |
| 3 | 15 (34.9%) | 8 (30.8%) | 7 (41.2%) |  |
| **Type of dignosis** |  |  |  | 0.646 |
| newly | 5 (10.2%) | 4 (12.5%) | 1 (5.88%) |  |
| recurrent | 44 (89.8%) | 28 (87.5%) | 16 (94.1%) |  |
| **Montreal classification of CD** | | | | |
| Montreal: A | |  |  | 0.042 |
| A1 | 4 (15.4%) | 2 (10.5%) | 2 (28.6%) |  |
| A2 | 14 (53.8%) | 13 (68.4%) | 1 (14.3%) |  |
| A3 | 8 (30.8%) | 4 (21.1%) | 4 (57.1%) |  |
| Montreal: L1~3 |  |  |  | 1.000 |
| L1 | 6 (23.1%) | 4 (21.1%) | 2 (28.6%) |  |
| L2 | 4 (15.4%) | 3 (15.8%) | 1 (14.3%) |  |
| L3 | 16 (61.5%) | 12 (63.2%) | 4 (57.1%) |  |
| Montreal: L4 |  |  |  | 0.540 |
| no | 21 (84.0%) | 15 (78.9%) | 6 (100%) |  |
| yes | 4 (16.0%) | 4 (21.1%) | 0 (0.00%) |  |
| Montreal: B |  |  |  | 0.857 |
| B1 | 8 (30.8%) | 5 (26.3%) | 3 (42.9%) |  |
| B2 | 10 (38.5%) | 8 (42.1%) | 2 (28.6%) |  |
| B3 | 8 (30.8%) | 6 (31.6%) | 2 (28.6%) |  |
| Montreal: Perianal lesion | |  |  | 0.006 |
| no | 14 (53.8%) | 7 (36.8%) | 7 (100%) |  |
| yes | 12 (46.2%) | 12 (63.2%) | 0 (0.00%) |  |
| **CDAI scores** | 4.75 (2.59) | 4.71 (2.80) | 4.86 (2.19) | 0.890 |
| **bestCDAI scores** | 197 (98.8) | 193 (92.0) | 208 (121) | 0.782 |
| **Disease severity of CD** | |  |  | 0.849 |
| mild | 7 (28.0%) | 6 (33.3%) | 1 (14.3%) |  |
| moderate | 11 (44.0%) | 7 (38.9%) | 4 (57.1%) |  |
| remission | 7 (28.0%) | 5 (27.8%) | 2 (28.6%) |  |
| **BMI, kg/m^2^** |  | 20.6 [17.9;23.0] | 19.9 [17.8;22.0] | 0.560 |
| **Medication history** | | | | |
| 5-ASA |  |  |  | 0.550 |
| no | 17 (23.0%) | 13 (26.0%) | 4 (16.7%) |  |
| yes | 57 (77.0%) | 37 (74.0%) | 20 (83.3%) |  |
| Steroid |  |  |  | 0.137 |
| no | 47 (61.8%) | 35 (68.6%) | 12 (48.0%) |  |
| yes | 29 (36.8%) | 16 (31.4%) | 13 (52.0%) |  |
| Immunosuppressant | |  |  | 0.526 |
| no | 63 (82.9%) | 41 (80.4%) | 22 (88.0%) |  |
| yes | 13 (17.1%) | 10 (19.6%) | 3 (12.0%) |  |
| TNF-αinhibitor |  |  |  | 1.000 |
| no | 65 (85.5%) | 43 (84.3%) | 22 (88.0%) |  |
| yes | 11 (14.5%) | 8 (15.7%) | 3 (12.0%) |  |
| Antibiotics |  |  |  | 0.366 |
| no | 62 (81.6%) | 40 (78.4%) | 22 (88.0%) |  |
| yes | 14 (18.4%) | 11 (21.6%) | 3 (12.0%) |  |
| PPI |  |  |  | 1.000 |
| no | 72 (94.7%) | 48 (94.1%) | 24 (96.0%) |  |
| yes | 4 (5.26%) | 3 (5.88%) | 1 (4.00%) |  |
| Parenteral nutrition | |  |  | 0.465 |
| no | 67 (88.2%) | 46 (90.2%) | 21 (84.0%) |  |
| yes | 9 (11.8%) | 5 (9.80%) | 4 (16.0%) |  |
| Enteral nutrition |  |  |  | 1.000 |
| no | 58 (76.3%) | 39 (76.5%) | 19 (76.0%) |  |
| yes | 18 (23.7%) | 12 (23.5%) | 6 (24.0%) |  |
| **Laboratory data** | | | | |
| WBC, ×10^9^/L | 6.79 (2.54) | 6.56 (2.65) | 7.29 (2.29) | 0.226 |
| HGB, mg/L | 114 (24.1) | 115 (26.7) | 112 (17.6) | 0.526 |
| PLT, ×10^9^/L | 304 [237;397] | 301 [226;389] | 316 [244;407] | 0.899 |
| ESR, mm/h | 18.0 [10.0;34.0] | 18.0 [11.5;37.0] | 18.0 [8.50;29.0] | 0.462 |
| hsCRP, mg/L | 8.44 [2.96;23.3] | 7.09 [2.63;26.1] | 11.2 [3.13;19.7] | 0.811 |
| Alb,×10^9^/L | 35.0 (6.22) | 35.2 (6.69) | 34.5 (5.18) | 0.599 |
| **CMV colitis** |  |  |  | 0.388 |
| no | 70 (92.1%) | 48 (94.1%) | 22 (88.0%) |  |
| yes | 6 (7.89%) | 3 (5.88%) | 3 (12.0%) |  |

**Supplementary Table 2.** Species list of the study groups

**Supplementary Table 3.** Taxonomy composition of the study groups

**Supplementary Table 4.** Microbial markers with significant differences among the study groups

**Supplementary Table 5.** Gut mycobiome analysis of the study groups

**Supplementary Table 6.** Functional analysis of the study groups

**Supplementary Table 7.** Network analysis of the study groups
